# Bis[2-(1*H*-benzotriazol-1-yl)-1*H*-benz­imidazol-1-ido]diethano­lcadmium

**DOI:** 10.1107/S1600536813001827

**Published:** 2013-02-02

**Authors:** Ping Cao, Jia-Cheng Liu, Dong-Cheng Hu

**Affiliations:** a College of Chemistry and Chemical Engineering, Key Laboratory of Eco-Environment-Related Polymer Materials of the Ministry of Education, Key Laboratory of Polymer Materials of Gansu Province, Key Laboratory of Bioelectrochemistry & Environmental Analysis of Gansu, Northwest Normal University, Lanzhou 730070, People’s Republic of China

## Abstract

In the title complex, [Cd(C_13_H_8_N_5_)_2_(C_2_H_5_OH)_2_], the Cd^II^ cation is located on an inversion center and coordinated by two deprotonated 2-(1*H*-benzotriazol-1-yl)-1*H*-benzimid­azol-1-ide (*L*) ligands and two ethanol mol­ecules in a distorted N_4_O_2_ octa­hedral geometry. In the *L* ligand, the dihedral angle between benzoimidazole and benzotriazole ring systems is 10.8 (3)°. In the crystal, the complex mol­ecules are connected by O—H⋯N hydrogen bonds; inter­molecular π–π stacking is also observed [centroid–centroid distances of 3.668 (5) Å between triazole and benzene rings and 3.780 (5) Å between imidazole rings].

## Related literature

For applications of metal comlexes with heterocyclic ligands, see: Zhou *et al.* (2006 ▶); Batten & Robson (1998 ▶); Zaworotko (1994 ▶). For a related structure, see: Wu *et al.* (2009 ▶).
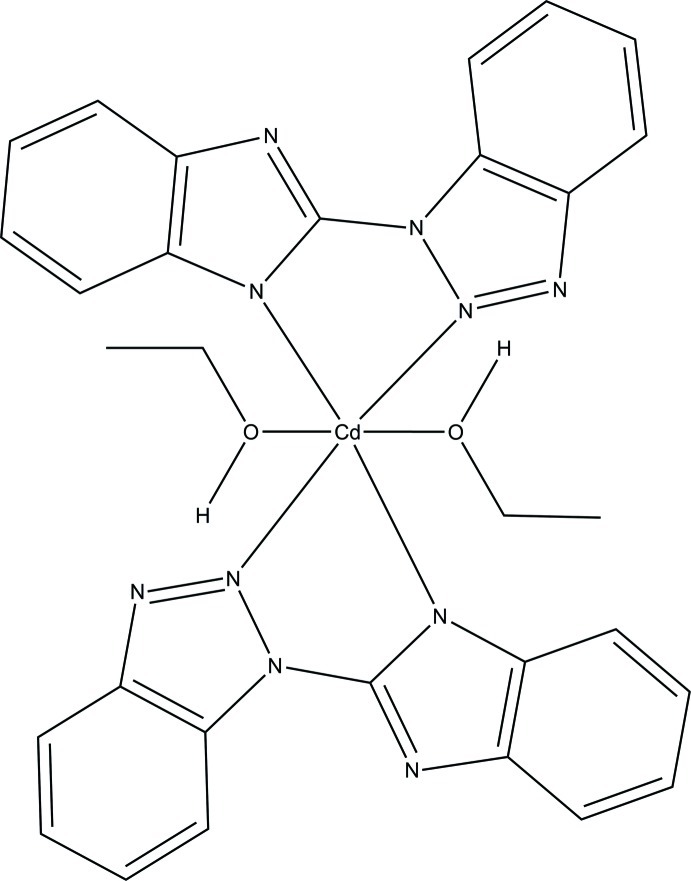


## Experimental

### 

#### Crystal data

[Cd(C_13_H_8_N_5_)_2_(C_2_H_6_O)_2_]
*M* *_r_* = 673.03
Monoclinic, 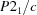

*a* = 8.7544 (4) Å
*b* = 8.0112 (2) Å
*c* = 20.9382 (9) Å
β = 101.352 (5)°
*V* = 1439.74 (10) Å^3^
*Z* = 2
Mo *K*α radiation
μ = 0.81 mm^−1^
*T* = 293 K
0.32 × 0.28 × 0.25 mm

#### Data collection

Agilent SuperNova (Dual, Cu at zero, Eos) diffractometer
Absorption correction: multi-scan (*CrysAlis PRO*; Agilent, 2012 ▶) *T*_min_ = 0.773, *T*_max_ = 0.818
7375 measured reflections
3636 independent reflections
2781 reflections with *I* > 2σ(*I*)
*R*_int_ = 0.030

#### Refinement

*R*[*F* ^2^ > 2σ(*F* ^2^)] = 0.089
*wR*(*F* ^2^) = 0.221
*S* = 1.20
3636 reflections
200 parameters
1 restraint
H atoms treated by a mixture of independent and constrained refinement
Δρ_max_ = 2.99 e Å^−3^
Δρ_min_ = −0.97 e Å^−3^

### 

Data collection: *CrysAlis PRO* (Agilent, 2012 ▶); cell refinement: *CrysAlis PRO*; data reduction: *CrysAlis PRO*; program(s) used to solve structure: *SHELXTL* (Sheldrick, 2008 ▶); program(s) used to refine structure: *SHELXTL*; molecular graphics: *SHELXTL*; software used to prepare material for publication: *SHELXTL*.

## Supplementary Material

Crystal structure: contains datablock(s) I, global. DOI: 10.1107/S1600536813001827/xu5670sup1.cif

Structure factors: contains datablock(s) I. DOI: 10.1107/S1600536813001827/xu5670Isup2.hkl

Additional supplementary materials:  crystallographic information; 3D view; checkCIF report

## Figures and Tables

**Table 1:** Selected bond lengths (Å)

Cd1—O1	2.414 (7)
Cd1—N2	2.494 (7)
Cd1—N5	2.180 (6)

**Table 2:** Hydrogen-bond geometry (Å, °)

*D*—H⋯*A*	*D*—H	H⋯*A*	*D*⋯*A*	*D*—H⋯*A*
O1—H1⋯N4^i^	0.85 (1)	1.98 (5)	2.787 (9)	159 (12)

Symmetry code: (i) 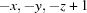.

**Fig. 1.**
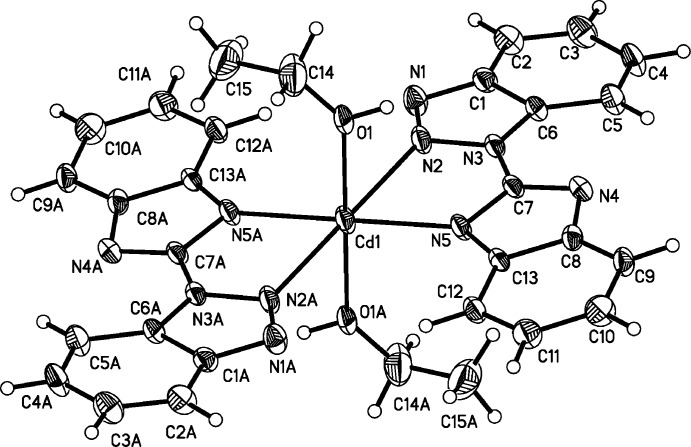
The molecular structure of the title compound with the atom numbering. Displacement ellipsoids are drawn at the 50% probability level.

**Fig. 2.**
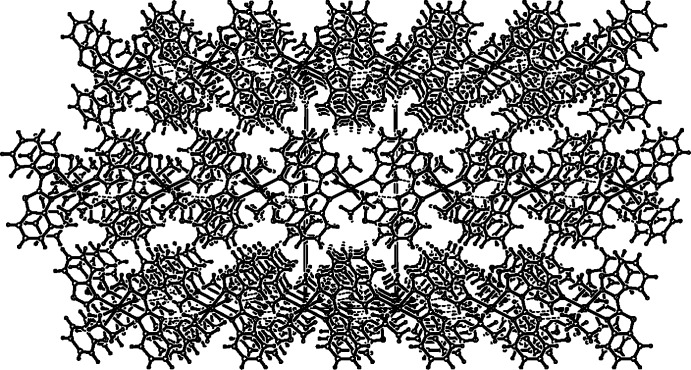
The crystal packing of the title compound, viewed along the a axis.

## supplementary crystallographic information

### Comment

Over the past decades, we lay much stress on the complexation of metal ions by nitrogen heterocyclic compounds as their applications in the areas of optical, lectronic properties and magnetice (Zhou *et al.*, 2006; Batten & Robson, 1998; Zaworotko, 1994).

The title compound possesses the benzotriazole and the benzimidazole rings and can offer possibilities to form complicated coordination complexes (Wu *et al.* 2009). In the crystal, the asymmetric unit contains one half Cd^2+^ cation, one organic *L* ligands and one ethanol molecules. The Cd^2+^ is coordinated by four N atoms from two different *L* ligands and two O atoms from two ethanol molecules. Molecules are connected by O—H···N hydrogen bonds and π-π interactions [centroid–centroid distance = 3.668 (5) and 3.780 (5) Å] involving related triazole, imidazole and benzene rings.

### Experimental

To a yellow solution of *L* (35 mg, 0.15 mmol) and Cd(NO_3_)_2_ (52.3 mg, 0.3 mmol) in ethanol (15 ml) were placed in a Teflon lined stainless steel autoclave and heated at 120 °C for 3 days under autogenous pressure. Then it was allowed to cool to room temperature. Stick-shaped crystals were collected in 50% yield. The crystals were repeatedly washed with ethanol and air-dried.

### Refinement

Ethanol H atom was located in a difference Fourier map and positional parameters were refined, U_iso_(H) = 1.5U_eq_(O). The C-bound H atoms were included in calculated position and refined in riding-model approximation with C—H = 0.93 Å, *U*_iso_(H)= 1.2*U*_eq_(C).

### Crystal data

**Table:**

[Cd(C_13_H_8_N_5_)_2_(C_2_H_6_O)_2_]	*F*(000) = 684
*M**_r_* = 673.03	*D*_x_ = 1.552 Mg m^−^^3^
Monoclinic, *P*2_1_/*c*	Mo *K*α radiation, λ = 0.71073 Å
Hall symbol: -P 2ybc	Cell parameters from 3469 reflections
*a* = 8.7544 (4) Å	θ = 3.4–28.4°
*b* = 8.0112 (2) Å	µ = 0.81 mm^−^^1^
*c* = 20.9382 (9) Å	*T* = 293 K
β = 101.352 (5)°	Block, yellow
*V* = 1439.74 (10) Å^3^	0.32 × 0.28 × 0.25 mm
*Z* = 2	

### Data collection

**Table:**

Agilent SuperNova (Dual, Cu at zero, Eos) diffractometer	3636 independent reflections
Radiation source: fine-focus sealed tube	2781 reflections with *I* > 2σ(*I*)
Graphite monochromator	*R*_int_ = 0.030
Detector resolution: 16.0733 pixels mm^-1^	θ_max_ = 28.5°, θ_min_ = 3.4°
ω scans	*h* = −11→11
Absorption correction: multi-scan (*CrysAlis PRO*; Agilent, 2012)	*k* = −9→10
*T*_min_ = 0.773, *T*_max_ = 0.818	*l* = −26→28
7375 measured reflections	

### Refinement

**Table:**

Refinement on *F*^2^	Primary atom site location: structure-invariant direct methods
Least-squares matrix: full	Secondary atom site location: difference Fourier map
*R*[*F*^2^ > 2σ(*F*^2^)] = 0.089	Hydrogen site location: inferred from neighbouring sites
*wR*(*F*^2^) = 0.221	H atoms treated by a mixture of independent and constrained refinement
*S* = 1.20	*w* = 1/[σ^2^(*F*_o_^2^) + (0.0356*P*)^2^ + 19.0636*P*] where *P* = (*F*_o_^2^ + 2*F*_c_^2^)/3
3636 reflections	(Δ/σ)_max_ = 0.001
200 parameters	Δρ_max_ = 2.99 e Å^−^^3^
1 restraint	Δρ_min_ = −0.97 e Å^−^^3^

### Special details

**Table:**

Geometry. All e.s.d.'s (except the e.s.d. in the dihedral angle between two l.s. planes) are estimated using the full covariance matrix. The cell e.s.d.'s are taken into account individually in the estimation of e.s.d.'s in distances, angles and torsion angles; correlations between e.s.d.'s in cell parameters are only used when they are defined by crystal symmetry. An approximate (isotropic) treatment of cell e.s.d.'s is used for estimating e.s.d.'s involving l.s. planes.
Refinement. Refinement of *F*^2^ against ALL reflections. The weighted *R*-factor *wR* and goodness of fit *S* are based on *F*^2^, conventional *R*-factors *R* are based on *F*, with *F* set to zero for negative *F*^2^. The threshold expression of *F*^2^ > σ(*F*^2^) is used only for calculating *R*-factors(gt) *etc*. and is not relevant to the choice of reflections for refinement. *R*-factors based on *F*^2^ are statistically about twice as large as those based on *F*, and *R*- factors based on ALL data will be even larger.

### Fractional atomic coordinates and isotropic or equivalent isotropic displacement parameters (Å^2^)

**Table:**

	*x*	*y*	*z*	*U*_iso_*/*U*_eq_	
Cd1	0.0000	0.5000	0.5000	0.0382 (3)	
N5	0.0518 (8)	0.2674 (8)	0.4545 (3)	0.0345 (15)	
N4	0.1590 (8)	0.0074 (9)	0.4596 (3)	0.0371 (15)	
C7	0.1439 (9)	0.1508 (9)	0.4883 (4)	0.0303 (15)	
N2	0.1878 (9)	0.3336 (8)	0.5803 (3)	0.0397 (16)	
C4	0.5416 (12)	−0.0727 (12)	0.6419 (5)	0.049 (2)	
H4	0.6007	−0.1689	0.6413	0.059*	
C6	0.3410 (9)	0.1084 (10)	0.5935 (4)	0.0341 (17)	
C5	0.4252 (11)	−0.0382 (11)	0.5883 (5)	0.047 (2)	
H5	0.4046	−0.1068	0.5518	0.057*	
C3	0.5740 (11)	0.0307 (12)	0.6967 (5)	0.050 (2)	
H3	0.6556	0.0032	0.7307	0.060*	
C13	−0.0046 (9)	0.1884 (9)	0.3956 (4)	0.0320 (16)	
O1	−0.2074 (8)	0.3361 (7)	0.5291 (4)	0.0470 (16)	
H1	−0.199 (14)	0.233 (3)	0.522 (6)	0.071*	
N3	0.2245 (8)	0.1886 (8)	0.5512 (3)	0.0317 (14)	
C9	0.0275 (11)	−0.0769 (11)	0.3455 (4)	0.0404 (19)	
H9	0.0686	−0.1842	0.3475	0.048*	
C14	−0.3409 (15)	0.3597 (16)	0.5541 (8)	0.081 (4)	
H14A	−0.3628	0.2586	0.5762	0.098*	
H14B	−0.4281	0.3800	0.5185	0.098*	
C2	0.4898 (11)	0.1690 (12)	0.7015 (5)	0.046 (2)	
H2	0.5096	0.2352	0.7387	0.055*	
C12	−0.1048 (11)	0.2473 (11)	0.3394 (4)	0.042 (2)	
H12	−0.1483	0.3536	0.3372	0.050*	
C10	−0.0704 (12)	−0.0196 (14)	0.2903 (5)	0.052 (2)	
H10	−0.0937	−0.0885	0.2539	0.063*	
C11	−0.1348 (12)	0.1384 (12)	0.2877 (5)	0.049 (2)	
H11	−0.2010	0.1723	0.2496	0.059*	
C8	0.0624 (9)	0.0309 (9)	0.3979 (4)	0.0326 (17)	
C1	0.3704 (10)	0.2104 (10)	0.6481 (4)	0.0361 (17)	
N1	0.2753 (9)	0.3473 (9)	0.6380 (4)	0.0430 (17)	
C15	−0.3265 (19)	0.5020 (19)	0.6007 (8)	0.096 (5)	
H15A	−0.3235	0.6049	0.5774	0.144*	
H15B	−0.2324	0.4903	0.6328	0.144*	
H15C	−0.4145	0.5027	0.6218	0.144*	

### Atomic displacement parameters (Å^2^)

**Table:**

	*U*^11^	*U*^22^	*U*^33^	*U*^12^	*U*^13^	*U*^23^
Cd1	0.0529 (5)	0.0192 (4)	0.0413 (5)	0.0066 (4)	0.0064 (4)	−0.0031 (4)
N5	0.046 (4)	0.017 (3)	0.039 (4)	0.004 (3)	0.004 (3)	0.001 (3)
N4	0.045 (4)	0.026 (3)	0.041 (4)	0.006 (3)	0.008 (3)	0.007 (3)
C7	0.035 (4)	0.018 (3)	0.038 (4)	0.000 (3)	0.008 (3)	0.004 (3)
N2	0.057 (4)	0.022 (3)	0.039 (4)	0.010 (3)	0.006 (3)	−0.003 (3)
C4	0.058 (6)	0.030 (4)	0.053 (6)	0.013 (4)	−0.005 (4)	0.003 (4)
C6	0.041 (4)	0.020 (3)	0.041 (4)	0.000 (3)	0.010 (3)	0.002 (3)
C5	0.055 (5)	0.032 (5)	0.053 (5)	0.009 (4)	0.005 (4)	0.001 (4)
C3	0.049 (5)	0.043 (6)	0.052 (5)	0.001 (4)	−0.006 (4)	0.004 (4)
C13	0.043 (4)	0.020 (3)	0.033 (4)	0.005 (3)	0.007 (3)	0.004 (3)
O1	0.064 (4)	0.019 (3)	0.064 (4)	0.007 (3)	0.027 (3)	−0.001 (3)
N3	0.044 (4)	0.019 (3)	0.032 (3)	0.003 (3)	0.008 (3)	0.000 (3)
C9	0.057 (5)	0.027 (4)	0.035 (4)	0.009 (4)	0.004 (4)	−0.007 (3)
C14	0.070 (8)	0.053 (7)	0.130 (12)	0.010 (6)	0.042 (8)	−0.007 (8)
C2	0.054 (5)	0.039 (5)	0.040 (5)	−0.004 (4)	−0.003 (4)	−0.002 (4)
C12	0.059 (5)	0.029 (4)	0.035 (4)	0.013 (4)	0.005 (4)	0.004 (3)
C10	0.066 (6)	0.049 (6)	0.038 (5)	−0.003 (5)	0.001 (4)	−0.008 (5)
C11	0.061 (6)	0.045 (5)	0.036 (5)	0.003 (5)	−0.001 (4)	−0.001 (4)
C8	0.037 (4)	0.024 (4)	0.037 (4)	0.001 (3)	0.008 (3)	−0.005 (3)
C1	0.046 (4)	0.028 (4)	0.033 (4)	−0.001 (3)	0.005 (3)	0.000 (3)
N1	0.056 (4)	0.032 (4)	0.035 (4)	0.007 (3)	−0.004 (3)	−0.007 (3)
C15	0.122 (12)	0.065 (8)	0.120 (12)	−0.018 (9)	0.074 (10)	−0.029 (9)

### Geometric parameters (Å, º)

**Table:**

Cd1—O1^i^	2.414 (7)	C13—C8	1.388 (10)
Cd1—O1	2.414 (7)	C13—C12	1.404 (11)
Cd1—N2	2.494 (7)	O1—C14	1.384 (13)
Cd1—N2^i^	2.494 (7)	O1—H1	0.848 (10)
Cd1—N5^i^	2.180 (6)	C9—C10	1.375 (13)
Cd1—N5	2.180 (6)	C9—C8	1.382 (11)
N5—C7	1.341 (10)	C9—H9	0.9300
N5—C13	1.388 (10)	C14—C15	1.489 (18)
N4—C7	1.316 (10)	C14—H14A	0.9700
N4—C8	1.411 (10)	C14—H14B	0.9700
C7—N3	1.399 (10)	C2—C1	1.411 (12)
N2—N1	1.301 (10)	C2—H2	0.9300
N2—N3	1.380 (9)	C12—C11	1.374 (13)
C4—C5	1.387 (13)	C12—H12	0.9300
C4—C3	1.398 (14)	C10—C11	1.383 (14)
C4—H4	0.9300	C10—H10	0.9300
C6—N3	1.371 (10)	C11—H11	0.9300
C6—C1	1.388 (11)	C1—N1	1.368 (11)
C6—C5	1.402 (11)	C15—H15A	0.9600
C5—H5	0.9300	C15—H15B	0.9600
C3—C2	1.346 (13)	C15—H15C	0.9600
C3—H3	0.9300		
			
N5^i^—Cd1—N5	180.000 (1)	C8—C13—N5	108.1 (7)
N5^i^—Cd1—O1^i^	82.9 (2)	C8—C13—C12	121.6 (8)
N5—Cd1—O1^i^	97.1 (2)	N5—C13—C12	130.2 (7)
N5^i^—Cd1—O1	97.1 (2)	C14—O1—Cd1	138.9 (7)
N5—Cd1—O1	82.9 (2)	C14—O1—H1	108 (8)
O1^i^—Cd1—O1	180.0 (2)	Cd1—O1—H1	113 (8)
N5^i^—Cd1—N2	109.2 (2)	C6—N3—N2	108.5 (6)
N5—Cd1—N2	70.8 (2)	C6—N3—C7	132.8 (7)
O1^i^—Cd1—N2	91.8 (2)	N2—N3—C7	118.7 (6)
O1—Cd1—N2	88.2 (2)	C10—C9—C8	117.5 (8)
N5^i^—Cd1—N2^i^	70.8 (2)	C10—C9—H9	121.2
N5—Cd1—N2^i^	109.2 (2)	C8—C9—H9	121.2
O1^i^—Cd1—N2^i^	88.2 (2)	O1—C14—C15	112.6 (11)
O1—Cd1—N2^i^	91.8 (2)	O1—C14—H14A	109.1
N2—Cd1—N2^i^	180.0 (3)	C15—C14—H14A	109.1
C7—N5—C13	102.9 (6)	O1—C14—H14B	109.1
C7—N5—Cd1	121.3 (5)	C15—C14—H14B	109.1
C13—N5—Cd1	135.2 (5)	H14A—C14—H14B	107.8
C7—N4—C8	101.9 (6)	C3—C2—C1	117.8 (9)
N4—C7—N5	118.1 (7)	C3—C2—H2	121.1
N4—C7—N3	122.8 (7)	C1—C2—H2	121.1
N5—C7—N3	119.1 (7)	C11—C12—C13	115.9 (8)
N1—N2—N3	109.6 (6)	C11—C12—H12	122.0
N1—N2—Cd1	140.4 (5)	C13—C12—H12	122.0
N3—N2—Cd1	109.4 (5)	C9—C10—C11	121.4 (9)
C5—C4—C3	123.0 (9)	C9—C10—H10	119.3
C5—C4—H4	118.5	C11—C10—H10	119.3
C3—C4—H4	118.5	C12—C11—C10	122.5 (9)
N3—C6—C1	104.5 (7)	C12—C11—H11	118.7
N3—C6—C5	132.7 (8)	C10—C11—H11	118.7
C1—C6—C5	122.8 (8)	C9—C8—C13	121.0 (8)
C4—C5—C6	114.7 (9)	C9—C8—N4	130.0 (7)
C4—C5—H5	122.7	C13—C8—N4	109.0 (7)
C6—C5—H5	122.7	N1—C1—C6	109.6 (7)
C2—C3—C4	121.5 (9)	N1—C1—C2	130.1 (8)
C2—C3—H3	119.2	C6—C1—C2	120.2 (8)
C4—C3—H3	119.2	N2—N1—C1	107.8 (7)
			
N5^i^—Cd1—N5—C7	32 (100)	N2^i^—Cd1—O1—C14	−59.3 (13)
O1^i^—Cd1—N5—C7	93.0 (6)	C1—C6—N3—N2	0.3 (9)
O1—Cd1—N5—C7	−87.0 (6)	C5—C6—N3—N2	−177.3 (9)
N2—Cd1—N5—C7	3.5 (6)	C1—C6—N3—C7	180.0 (8)
N2^i^—Cd1—N5—C7	−176.5 (6)	C5—C6—N3—C7	2.4 (15)
N5^i^—Cd1—N5—C13	−158 (100)	N1—N2—N3—C6	0.1 (9)
O1^i^—Cd1—N5—C13	−97.4 (8)	Cd1—N2—N3—C6	173.1 (5)
O1—Cd1—N5—C13	82.6 (8)	N1—N2—N3—C7	−179.6 (7)
N2—Cd1—N5—C13	173.2 (8)	Cd1—N2—N3—C7	−6.6 (8)
N2^i^—Cd1—N5—C13	−6.8 (8)	N4—C7—N3—C6	7.9 (13)
C8—N4—C7—N5	−0.4 (9)	N5—C7—N3—C6	−169.3 (8)
C8—N4—C7—N3	−177.7 (7)	N4—C7—N3—N2	−172.5 (7)
C13—N5—C7—N4	1.5 (10)	N5—C7—N3—N2	10.3 (11)
Cd1—N5—C7—N4	174.0 (5)	Cd1—O1—C14—C15	−33 (2)
C13—N5—C7—N3	178.8 (7)	C4—C3—C2—C1	−2.2 (15)
Cd1—N5—C7—N3	−8.6 (10)	C8—C13—C12—C11	1.5 (13)
N5^i^—Cd1—N2—N1	−8.7 (10)	N5—C13—C12—C11	178.0 (9)
N5—Cd1—N2—N1	171.3 (10)	C8—C9—C10—C11	−1.7 (15)
O1^i^—Cd1—N2—N1	74.5 (10)	C13—C12—C11—C10	−0.3 (15)
O1—Cd1—N2—N1	−105.5 (10)	C9—C10—C11—C12	0.4 (17)
N2^i^—Cd1—N2—N1	130 (100)	C10—C9—C8—C13	2.8 (13)
N5^i^—Cd1—N2—N3	−178.2 (5)	C10—C9—C8—N4	−179.3 (9)
N5—Cd1—N2—N3	1.8 (5)	N5—C13—C8—C9	180.0 (8)
O1^i^—Cd1—N2—N3	−95.1 (5)	C12—C13—C8—C9	−2.8 (13)
O1—Cd1—N2—N3	84.9 (5)	N5—C13—C8—N4	1.7 (9)
N2^i^—Cd1—N2—N3	−39 (100)	C12—C13—C8—N4	178.9 (8)
C3—C4—C5—C6	−0.5 (15)	C7—N4—C8—C9	−178.9 (9)
N3—C6—C5—C4	176.7 (9)	C7—N4—C8—C13	−0.8 (9)
C1—C6—C5—C4	−0.6 (13)	N3—C6—C1—N1	−0.7 (9)
C5—C4—C3—C2	2.1 (17)	C5—C6—C1—N1	177.3 (8)
C7—N5—C13—C8	−1.9 (9)	N3—C6—C1—C2	−177.6 (8)
Cd1—N5—C13—C8	−172.8 (6)	C5—C6—C1—C2	0.4 (13)
C7—N5—C13—C12	−178.7 (9)	C3—C2—C1—N1	−175.1 (9)
Cd1—N5—C13—C12	10.3 (14)	C3—C2—C1—C6	1.1 (14)
N5^i^—Cd1—O1—C14	11.6 (13)	N3—N2—N1—C1	−0.5 (10)
N5—Cd1—O1—C14	−168.4 (13)	Cd1—N2—N1—C1	−170.1 (7)
O1^i^—Cd1—O1—C14	−110 (100)	C6—C1—N1—N2	0.7 (10)
N2—Cd1—O1—C14	120.7 (13)	C2—C1—N1—N2	177.2 (9)

Symmetry code: (i) −*x*, −*y*+1, −*z*+1.

### Hydrogen-bond geometry (Å, º)

**Table:**

*D*—H···*A*	*D*—H	H···*A*	*D*···*A*	*D*—H···*A*
O1—H1···N4^ii^	0.85 (1)	1.98 (5)	2.787 (9)	159 (12)

Symmetry code: (ii) −*x*, −*y*, −*z*+1.
